# Hepatocyte Toll-Like Receptor 4 Mediates Alcohol-Induced Insulin Resistance in Mice

**DOI:** 10.3390/biom13030454

**Published:** 2023-03-01

**Authors:** Piumi B. Wickramasinghe, Shuwen Qian, Lyndsey E. Langley, Chen Liu, Lin Jia

**Affiliations:** 1 Department of Biological Sciences, The University of Texas at Dallas, Richardson, TX 75080, USA; 2 Center for Hypothalamic Research, Department of Internal Medicine, University of Texas Southwestern Medical Center, Dallas, TX 75390, USA

**Keywords:** alcohol, toll-like receptor 4, insulin resistance, hepatocytes

## Abstract

Accumulating evidence has demonstrated the association between alcohol overconsumption and the development of insulin resistance. However, the underlying mechanisms are not completely understood. To investigate the requirement and sufficiency of hepatocyte toll-like receptor 4 (TLR4) in alcohol-induced insulin resistance, we used two mouse models (Tlr4^fl/fl^ and Tlr4^LoxTB^) that allow ablation of TLR4 only in hepatocytes (Tlr4^LKO^) and restoration of endogenous TLR4 expression in hepatocytes on a TLR4-null background (Tlr4^LoxTB^ × Alb-Cre), respectively. A Lieber-DeCarli feeding model was used to induce glucose intolerance and insulin resistance in mice. Glucose tolerance test, insulin tolerance test, and insulin signaling experiments were performed to examine systemic and tissue-specific insulin sensitivity. We found that alcohol-fed hepatocyte TLR4 deficient mice (Tlr4^LKO^) had lower blood glucose levels in response to intraperitoneal injection of insulin. Moreover, increased phosphorylation of glycogen synthase kinase-3β (GSK3β) was observed in the liver of Tlr4^LKO^ mice after chronic alcohol intake. In contrast, when hepatic TLR4 was reactivated in mice (Tlr4^LoxTB^ × Alb-Cre), alcohol feeding caused glucose intolerance in these mice compared with littermate controls (Tlr4^LoxTB^). In addition, AKT phosphorylation was dramatically reduced in the liver and epididymal white adipose tissue (eWAT) of alcohol-fed Tlr4^LoxTB^ × Alb-Cre mice, which was similar to that of mice with whole-body TLR4 reactivation (Tlr4^LoxTB^ × Zp3-Cre). Collectively, these findings suggest that hepatocyte TLR4 is both required and sufficient in the development of insulin resistance induced by alcohol overconsumption.

## 1. Introduction

Excessive alcohol intake has become a growing public health concern worldwide, which causes detrimental effects on various organs. Increasing lines of evidence indicate an association between heavy drinking and the development of insulin resistance in humans [1,2,3] and rodents [4,5,6,7,8]. Specifically, reduced insulin signaling and decreased glucose transport have been reported in various tissues and cell types that were exposed to alcohol, including the liver [7,8,9,10,11], white adipose tissue [12], as well as isolated hepatocytes [13], and adipocytes [14,15]. However, the underlying mechanisms by which alcohol drinking causes insulin resistance and glucose dysregulation are not completely understood.

Extensive studies have demonstrated that lipopolysaccharide (LPS) and its cell surface receptor, toll-like receptor 4 (TLR4) play critical roles in the development of alcohol-associated liver disease (ALD) [16,17,18]. In addition, the cell-type-specific role of TLR4 in alcohol-induced fatty liver disease has been reported. Unexpectedly, TLR4-expressing myeloid cells played a minimal role in regulating alcohol-induced fatty liver disease and adipose tissue inflammation [19,20]. Interestingly, alcohol-fed hepatocyte TLR4 deficient mice showed significantly reduced hepatic triglyceride content and decreased expression of inflammatory cytokines in the white adipose tissue [19]. However, it is largely unknown whether hepatocyte TLR4 contributes to the development of insulin resistance induced by chronic alcohol drinking.

Increasing evidence suggests that hepatocyte TLR4 plays an important role in regulating obesity-associated glucose dysregulation and insulin resistance in mice. For example, Uchimura et al. reported that the knockdown of hepatic TLR4 by siRNA significantly improved insulin resistance in high-fat diet (HFD)-fed mice [21]. Moreover, mice lacking TLR4 selectively in hepatocytes exhibited enhanced systemic insulin sensitivity and reduced hepatic glucose production after HFD feeding [22]. These findings led us to investigate if hepatocyte TLR4 regulates alcohol-induced insulin resistance.

In the current study, hepatocyte TLR4 ablated mice and their littermate controls were fed either a control or alcohol-containing liquid diet chronically for 8 weeks. During the diet feeding, systemic and tissue-specific insulin sensitivity was examined. We found that hepatocyte TLR4 is required in mediating insulin resistance following excessive alcohol intake. This is supported by the observation that mice lacking hepatocyte TLR4 showed improved systemic insulin sensitivity and significantly increased protein expression of phosphorylated glycogen synthase kinase-3β (GSK3β) in the liver after chronic alcohol feeding. In addition, we generated mice with endogenous TLR4 restored in hepatocytes on a TLR4-null background. This mouse model allowed us to investigate the sufficiency of hepatocyte TLR4 in alcohol-induced impairment in insulin signaling. Our data showed that reactivation of hepatocyte TLR4 exacerbated alcohol-induced glucose intolerance and decreased insulin signaling. Collectively, these findings indicate that hepatocyte TLR4 plays a role in regulating alcohol-induced insulin resistance in mice.

## 2. Materials and Methods

### 2.1. Animal Care and Chronic Liquid Diet Feeding

The generation and validation of Tlr4^fl/fl^ [19,22,23] and Tlr4^LoxTB^ [24,25,26] mice have been previously reported. Albumin-Cre transgenic mice (Alb-Cre, JAX:003574) were crossed with Tlr4^fl/fl^ animals to produce mice lacking TLR4 in hepatocytes (Tlr4^LKO^) [19,24]. In addition, Alb-Cre and zona pellucida 3 (Zp3)-Cre mice (JAX:003651) were bred with Tlr4^LoxTB^ mice to restore endogenous TLR4 expression in hepatocytes (Tlr4^LoxTB^ × Alb-Cre) and in all tissues (Tlr4^LoxTB^ × Zp3-Cre) [24], respectively. All strains were backcrossed at least six generations to maintain C57BL/6J genetic background. Animals were housed (*n* = 4 per cage) in a temperature-controlled environment on a 12 h light/12 h dark cycle with ad libitum access to food and water unless specified otherwise.

Here, 10–12-week-old male mice were subjected to a control liquid diet (Bio-Serv, F1259SP) for five days. After the acclimation, mice were fed either control or alcohol-containing liquid diets (Bio-Serv, F1258SP, 5% alcohol (vol/vol)) for 8 weeks. Because mice consumed less of the alcohol-containing liquid diet than the control diet [27], mice on the control liquid diet were pair-fed. We recorded the food intake of the alcohol-fed mice based on the differences between the volume added the day before and the volume left. Then we calculated the average daily volume consumed by alcohol-fed mice. This calculated volume was the amount of control liquid diet given to pair-fed mice [27]. In addition, the dead volume on the bottom of the tubes was considered. An extra 5 mL of control liquid diet was added to the calculated volume to pair-fed mice. On the first day of liquid diet feeding, the volume of control liquid diets was estimated based on the pilot experiments and/or our previous experience. At the end of diet feeding, mice were fasted for 4 h. After deep anesthetization, blood was collected from the inferior vena cava, followed by an infusion of 10 mL PBS to the left ventricle of the heart to remove residual blood. Livers were removed and snap-frozen in liquid nitrogen and stored at −80 °C. Experiments were performed according to protocols reviewed and approved by the Institutional Animal Care and Use Committee of the University of Texas at Dallas (UTD).

### 2.2. Glucose and Insulin Tolerance Tests

A glucose tolerance test (GTT) was performed after 6 weeks of liquid diet feeding. The mice were fasted for 5 h. After the measurement of basal blood glucose levels with a glucometer (Contour, Bayer, Parsippany, NJ, USA), a glucose solution (1.2 g/kg body weight (BW)) was given via intraperitoneal injection. Then blood glucose concentration was measured 15, 30, 60, and 120 min after glucose administration. An insulin tolerance test (ITT) was performed after 7 weeks of diet feeding. Food was removed for 2 h in the morning, and blood glucose was measured before and 15, 30, 60, and 120 min after intraperitoneal injection of human insulin (1 unit/kg BW; Humulin R).

### 2.3. Acute Insulin Injection to Determine Tissue-Specific Insulin Sensitivity

After 8 weeks of alcohol-containing liquid diet feeding, mice were fasted for 6 h and injected intraperitoneally with either saline or human insulin (5 units/kg BW; Humulin R). 10 min later, liver and epididymal white adipose tissue (eWAT) were rapidly removed and snap-frozen in liquid nitrogen and stored at −80 °C until analysis.

### 2.4. Western Blotting

Liver and eWAT (pooled samples from the same genotype and treatment, *n* = 2–4) were homogenized in lysis buffer containing 1% NP-40, 1% Triton-X 100, 1% SDS, 5 mM EDTA (pH 8.0), 50 mM Tris– HCl (pH 7.4), and protease inhibitor (P8340, Sigma, St. Louis, MO, USA) and phosphatase inhibitor cocktails (P5726 and P0044, Sigma, St. Louis, MO, USA). Protein concentrations were determined by BCA kit (Pierce, Thermo Scientific™, Waltham, MA, USA). Then, 10–20 µg tissue lysates were separated by 8% gel in SDS-PAGE and transferred to nitrocellulose membranes (Trans-Blot, Bio-Rad, Hercules, CA, USA). Membranes were blocked with 5% nonfat dried milk or 3% BSA (for phosphorylated proteins) for 1 h at room temperature and then incubated with primary antibodies overnight at 4 °C in 5% nonfat dried milk or 3% BSA (for phosphorylated proteins). Primary antibodies were purchased from Cell Signaling Technology, phospho-AKT (Ser473) (#9271), phospho-GSK3β (#8466), total AKT (pan) (#4691), and total GSK3β (#9315). Membranes were washed with TBS containing 0.1% (vol/vol) Tween 20, and incubated with 1:10,000 dilution of goat anti-rabbit horseradish peroxidase antibody (Jackson ImmunoResearch, West Grove, PA, USA) for 1 h at room temperature. Blots were visualized with enhanced chemiluminescence (Bio-Rad, Hercules, CA, USA). The band densities were quantified using Image J software (NIH).

### 2.5. Blood Glucose and Plasma Insulin Levels

After 8 weeks of liquid diet feeding, blood was collected from 6 h fasted mice via tail bleeding. Blood glucose was measured using a glucometer (Contour, Bayer, Parsippany, NJ, USA). Plasma insulin concentration was determined using an ELISA (Crystal Chem, Elk Grove Village, IL, USA) according to the manufacturer’s instructions.

### 2.6. Measurement of Liver Triglyceride Contents

Frozen liver tissues (60–80 mg) were thawed, minced, and weighed in glass tubes. Lipids were extracted in 3 mL of chloroform/methanol (2:1) at room temperature overnight. After centrifugation, lipid extract was transferred to a clean glass tube, and dilute H_2_SO_4_ (0.05%) was added to separate the phases by vortex and centrifugation. The aqueous upper phase was removed, and an aliquot of the bottom phase (100 µL) was transferred to a new glass tube and dried down under N_2_. Then, 1 mL of 1% Triton X-100 in chloroform was added. After the evaporation of the solvent, deionized water (0.5 mL) was added to each tube and vortexed until the solution was clear. Triglyceride standards (Verichem Laboratories, Providence, RI, USA) was prepared by adding 1% Triton X-100 in chloroform, evaporating, and dissolving in deionized water. Triglyceride content in liver samples was quantified using the Infinity Triglycerides Reagent (Thermo Scientific™, Waltham, MA, USA).

### 2.7. Primary Hepatocyte Isolation and Real-Time PCR

Primary hepatocyte was isolated from chow-fed Tlr4^LoxTB^ and Tlr4^LoxTB^ × Alb-Cre mice as described previously [22]. Total RNAs from the hepatocytes were extracted using RNA STAT60 (Tel-Test, Friendswood, TX, USA). Complementary DNA was synthesized using the High Capacity cDNA Kit (Applied Biosystems, Waltham, MA, USA) and qPCR was performed using a Bio-Rad sequence detection system (Bio-Rad). Primers for Tlr4 (forward, CAGCAAAGTCCCTGATGACA and reverse, AGAGGTGGTGTAAGCCATGC) and 18s (forward, ACCGCAGCTAGGAATAATGGA and reverse, GCCTCAGTTCCGAAAACCA) were purchased from Integrated DNA Technologies. The relative amounts of Tlr4 mRNAs were calculated using the ΔΔCT assay.

### 2.8. Statistical Analysis

Data are expressed as means ± SEM. Statistical analysis was performed using a *t*-test when there was only one variable or analysis of variance (two-way ANOVA) with Post hoc Tukey’s multiple comparisons test or Šídák’s multiple comparisons tests when two variables (genotype and treatment) were present (GraphPad Prism, Boston, FL, USA). *p* < 0.05 is considered significant.

## 3. Results

### 3.1. Alcohol-Fed Hepatocyte TLR4 Deficient Mice Exhibited Improved Systemic Insulin Sensitivity

**Figure 1:**
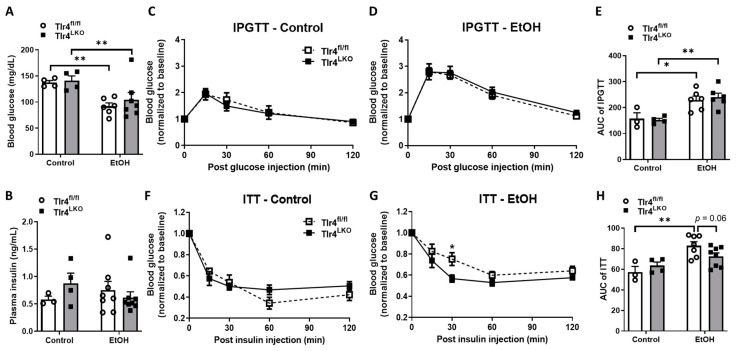
Systemic glucose tolerance and insulin sensitivity in Tlr4^LKO^ and littermate Tlr4^fl/fl^ mice after chronic liquid diet feeding. (**A**) Blood glucose (control treatment (*n* = 4) and EtOH treatment (*n* = 6–7)) and (**B**) plasma insulin (control treatment (*n* = 3–4) and EtOH treatment (*n* = 8)) levels in mice after a 6 h fast. (**C**,**D**) Intraperitoneal glucose tolerance tests (IPGTTs) in Tlr4^fl/fl^ and Tlr4^LKO^ mice after chronic control (**C**, *n* = 3–4) and alcohol-containing (**D**, *n* = 6) liquid diet feeding. IPGTTs were performed in 5 h fasted mice with intraperitoneal injection of glucose at 1.2 g/kg BW. Blood glucose levels were determined from tail bleeds at the indicated time points after glucose administration. Areas under the curve (AUC) were analyzed for IPGTTs (**E**). (**F**,**G**) Insulin tolerance tests (ITTs) in Tlr4^fl/fl^ and Tlr4^LKO^ mice after chronic control (**F**, *n* = 3–4) and alcohol-containing (**G**, *n* = 7–8) liquid diet feeding. ITTs were performed 2 h after food removal. Mice were injected intraperitoneally with insulin at 1.0 unit/kg BW. Blood glucose levels were determined from tail bleeds at the indicated time points by a glucometer (Baylor). Areas under the curve (AUC) were analyzed for ITTs (**H**). *, *p* < 0.05, **, *p* < 0.01, compared with Tlr4^fl/fl^ mice. All data are presented as mean ± SEM.

To examine whether hepatocyte TLR4 regulates chronic alcohol-drinking-associated glucose dysregulation and insulin insensitivity, both Tlr4^fl/fl^ and Tlr4^LKO^ mice were fed either a control or 5% alcohol-containing liquid diet chronically for 8 weeks. We found that Tlr4^fl/fl^ and Tlr4^LKO^ mice on the control diet exhibited similar blood glucose and plasma insulin levels after a 6 h fast (Figure 1A,B). Consistent with a previous report [28], excessive alcohol drinking led to reduced blood glucose levels in both genotypes (Figure 1A). However, fasting plasma insulin concentrations were not affected by alcohol feeding or hepatocyte TLR4 deficiency (Figure 1B). 

To determine the systemic glucose homeostasis and insulin sensitivity in these mice, glucose and insulin tolerance tests (IPGTT and ITT) were performed. After chronic control diet feeding, Tlr4^fl/fl^ and Tlr4^LKO^ mice responded similarly to glucose administration and insulin injection (Figure 1C,F). Chronic alcohol feeding caused glucose intolerance in both Tlr4^fl/fl^ and Tlr4^LKO^ mice (Figure 1D,E). Although alcohol-fed Tlr4^fl/fl^ mice developed systemic insulin resistance (Figure 1G,H), Tlr4^LKO^ mice were insulin sensitive after excessive alcohol drinking as evidenced by lower normalized blood glucose levels after 30 min of insulin injection (Figure 1G), which was comparable to mice pair-fed control liquid diet (Figure 1F,H). The analysis of the area under the curve (AUC) also showed that Tlr4^LKO^ mice had improved systemic insulin sensitivity after chronic alcohol feeding (Figure 1H).

### 3.2. Alcohol-Fed Hepatocyte TLR4 Deficient Mice Showed Improved Insulin Sensitivity in the Liver

**Figure 2:**
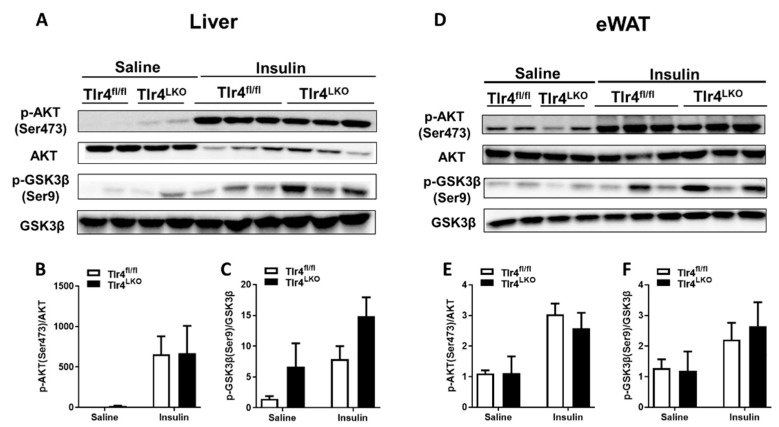
Western blot analysis of proteins involved in insulin signaling in the liver (**A**) and eWAT (**D**) of alcohol-fed Tlr4^fl/fl^ and Tlr4^LKO^ mice. After 8 weeks of chronic alcohol feeding, Tlr4^fl/fl^ and Tlr4^LKO^ mice were fasted for 6 h. Saline or 5 units/kg BW of recombinant human insulin (Humulin R) was injected intraperitoneally. Ten minutes later, liver and epididymal white adipose tissue (eWAT) were quickly removed and snap-frozen in liquid nitrogen. Antibodies were purchased from Cell Signaling Technology. Quantification of the band densities was expressed as fold changes of the phosphorylated form, normalized by total corresponding protein content, of AKT (Ser473) (**B**,**E**) and GSK3β (Ser9) (**C**,**F**) compared with those from saline-treated Tlr4^fl/fl^. All data are presented as mean ± SEM.

To examine the tissue-specific insulin sensitivity, Tlr4^fl/fl^ and Tlr4^LKO^ mice were fed an alcohol-containing liquid diet chronically for 8 weeks. Then saline or insulin was injected intraperitoneally after a 6 h fast. Liver whole cell lysates were prepared for the expression of proteins involved in the insulin signaling pathway. Regardless of the genotypes, insulin administration greatly elevated the expression of AKT phosphorylation of Ser473 (p-AKT on Ser473) compared with saline treatment (Figure 2A,B). Although comparable expression of total GSK3β was observed in saline and insulin-treated livers, phosphorylated GSK3β (p-GSK3β on Ser9) levels were higher in the hepatic whole cell lysates of alcohol-fed Tlr4^LKO^ mice (Figure 2A,C).

Impaired insulin signaling in epididymal white adipose tissue (eWAT) has been reported in alcohol-fed rodent models [12]. Therefore, Western blotting of total AKT and phosphorylated AKT (p-AKT) was performed on eWAT samples from mice fed an alcohol-containing liquid diet for 8 weeks. In response to insulin administration, similarly elevated AKT phosphorylation in the eWAT was observed in Tlr4^fl/fl^ and Tlr4^LKO^ mice (Figure 2D,E). Insulin stimulation of GSK phosphorylation on Ser9 in the eWAT tended to be increased in alcohol-fed Tlr4^LKO^ mice (Figure 2D). However, the densitometry quantification did not show a significant difference (Figure 2F). These findings suggest that hepatocyte TLR4 deficiency protects mice from chronic alcohol-induced insulin resistance in the liver.

### 3.3. Restoration of Endogenous TLR4 Expression in Hepatocytes Did Not Affect Blood Glucose and Plasma Insulin Levels after Chronic Alcohol Feeding

**Figure 3:**
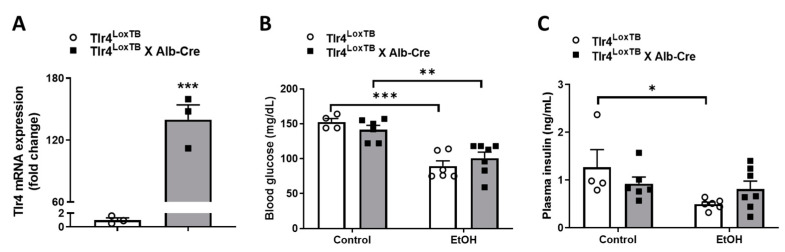
Alcohol-fed hepatocyte TLR4-reactivated mice showed reduced blood glucose levels. (**A**) qPCR analysis of TLR4 mRNA expression in primary hepatocytes isolated from chow-fed Tlr4^LoxTB^ × Alb-Cre (Ct value: 25.04 ± 0.50) and Tlr4^LoxTB^ mice (Ct value: 32.55 ± 0.85) (*n* = 3). Blood glucose (**B**, control treatment (*n* = 4–6) and EtOH treatment (*n* = 6–7)) and plasma insulin (**C**, control treatment (*n* = 4–6) and EtOH treatment (*n* = 6–7)) levels were determined in 6 h fasted mice after chronic liquid diet feeding. *, *p* < 0.05, **, *p* < 0.01, ***, *p* < 0.001, compared with Tlr4^LoxTB^ mice. Data are expressed as mean ± SEM.

To investigate the sufficiency of hepatocyte TLR4 in alcohol-induced insulin resistance, we used a mouse model that is conditionally null for TLR4 (Tlr4^LoxTB^) [24]. The utility of this mouse model has been widely tested [24,25,26]. To re-express Tlr4 selectively in hepatocytes, we crossed the Tlr4^LoxTB^ mice to Alb-Cre mice (Tlr4^LoxTB^ × Alb-Cre). As shown in Figure 3A, primary hepatocytes isolated from Tlr4^LoxTB^ × Alb-Cre mice had dramatically elevated endogenous TLR4 mRNA expression.

Then blood glucose and plasma insulin levels were determined in fasted Tlr4^LoxTB^ × Alb-Cre and littermate Tlr4^LoxTB^ mice after chronic liquid diet feeding. We found that alcohol-fed Tlr4^LoxTB^ and Tlr4^LoxTB^ × Alb-Cre mice showed similarly reduced blood glucose levels (Figure 3B). In addition, there were no differences in plasma insulin levels between Tlr4^LoxTB^ and Tlr4^LoxTB^ × Alb-Cre littermates fed a control liquid diet (Figure 3C). After chronic alcohol feeding, reduced plasma insulin concentrations were observed in Tlr4^LoxTB^ mice, but not in Tlr4^LoxTB^ × Alb-Cre littermates (Figure 3C).

### 3.4. Restoration of Endogenous TLR4 Expression in Hepatocytes Exacerbated Glucose Intolerance in Alcohol-Fed Mice

**Figure 4:**
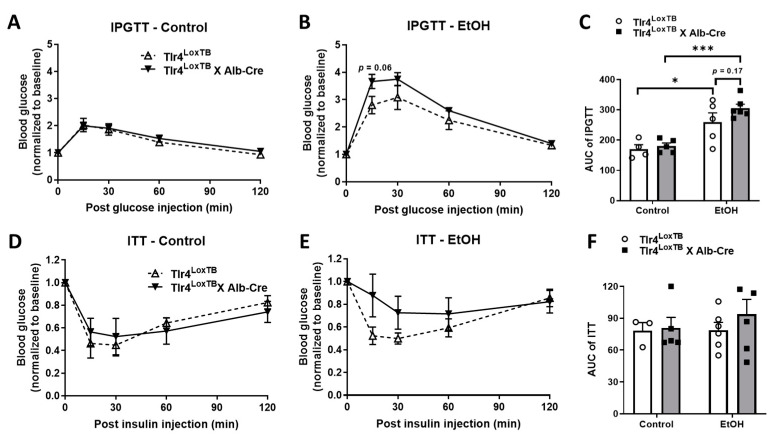
Reactivation of hepatocyte TLR4 tended to exacerbate systemic glucose intolerance in mice. (**A**,**B**) Intraperitoneal glucose tolerance tests (IPGTTs) were performed in mice after 6 weeks of either control (**A**, *n* = 4–5) or alcohol drinking (**B**, *n* = 5–8). Mice were fasted for 6 h and treated with 1.2 g/kg BW glucose intraperitoneally. Blood glucose levels were monitored before and 15, 30, 60, and 120 min after glucose injection. (**D**,**E**) For insulin tolerance tests (ITTs), mice were fasted for 2 h in the morning after 7 weeks of control (**D**, *n* = 4–5) or alcohol-containing (**E**, *n* = 5–6) liquid diet feeding. 1.0 unit/kg BW of insulin was injected via peritoneal cavity and blood glucose levels were measured by a glucometer. Areas under the curve (AUC) were analyzed for IPGTTs (**C**) and ITTs (**F**). *, *p* < 0.05, ***, *p* < 0.001, compared between mice of the same genotype fed different diets. All data are presented as mean ± SEM.

Next, we performed IPGTTs and ITTs in Tlr4^LoxTB^ and Tlr4^LoxTB^ × Alb-Cre mice after chronic liquid diet feeding. On control liquid diets, both genotypes showed similar blood glucose responses after intraperitoneal injection of glucose (Figure 4A,C). After chronic alcohol feeding, Tlr4^LoxTB^ mice had elevated normalized blood glucose levels, indicating glucose intolerance in these mice (Figure 4B,C). Interestingly, compared with Tlr4^LoxTB^ mice, alcohol-fed Tlr4^LoxTB^ × Alb-Cre mice exhibited even higher normalized blood glucose levels observed 15 min after glucose administration (Figure 4B). In addition, the AUC analysis showed that reactivation of TLR4 in hepatocytes tended to reduce systemic glucose tolerance in mice after chronic alcohol feeding (Figure 4C).

For ITTs, Tlr4^LoxTB^ mice maintained similar blood glucose levels on both control and alcohol-containing diets, suggesting that whole-body TLR4 knockout mice were resistant to alcohol-induced systemic insulin resistance (Figure 4D,F). After chronic alcohol feeding, Tlr4^LoxTB^ × Alb-Cre mice tended to have elevated blood glucose levels 15 and 30 min after insulin injection compared with Tlr4^LoxTB^ mice (Figure 4E). However, the increases were not significantly different (Figure 4E,F). These findings suggest that reactivation of hepatocyte TLR4 promotes alcohol-induced glucose intolerance in mice.

### 3.5. Restoration of Hepatocyte TLR4 in Mice Led to Impaired Phosphorylation of AKT in Both Liver and Adipose Tissue

**Figure 5:**
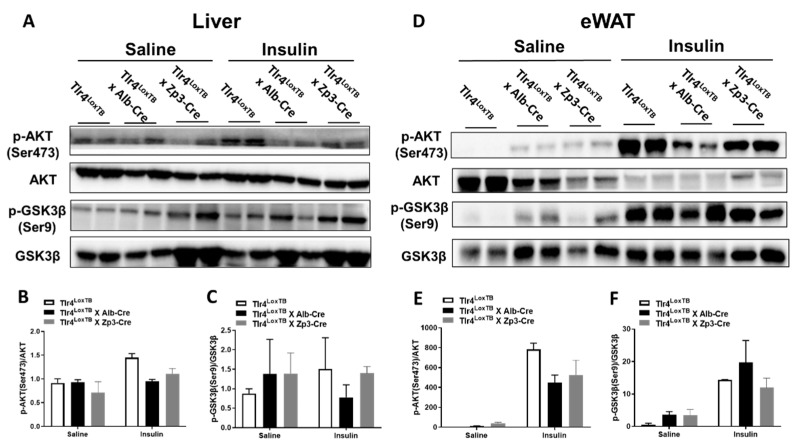
Western blot analysis of proteins involved in insulin signaling in the liver (**A**) and eWAT (**D**) of alcohol-fed Tlr4^LoxTB^, Tlr4^LoxTB^ × Alb-Cre and Tlr4^LoxTB^ × Zp3-Cre mice. After 8 weeks of chronic alcohol feeding, mice were fasted for 6 h. Saline or 5 units/kg BW of recombinant human insulin (Humulin R) was injected intraperitoneally. Ten minutes later, liver and epididymal white adipose tissue (eWAT) were quickly removed and snap-frozen in liquid nitrogen. Antibodies were purchased from Cell Signaling Technology. Quantification of the band densities was expressed as fold changes of the phosphorylated form, normalized by total corresponding protein content, of AKT (Ser473) (**B**,**E**) and GSK3β (Ser9) (**C**,**F**) compared with those from saline-treated Tlr4^LoxTB^. All data are presented as mean ± SEM.

To determine whether TLR4 reactivation in hepatocytes is sufficient to impair insulin signaling in the liver and adipose tissue, we performed acute saline and insulin injection in alcohol-fed Tlr4^LoxTB^, Tlr4^LoxTB^ × Alb-Cre, and Tlr4^LoxTB^ × Zp3-Cre mice. Compared to saline treatment, insulin administration caused increased AKT phosphorylation of Ser473 (p-AKT on Ser473) in the liver of Tlr4^LoxTB^ mice (Figure 5A,B). Interestingly, this increase was dramatically blunted in livers prepared from Tlr4^LoxTB^ × Alb-Cre mice. However, restoration of TLR4 in hepatocytes and in the whole body did not suppress hepatic p-GSK3β expression (Figure 5A,C). In addition, western blot analyses were performed in whole cell lysates of eWAT from saline and insulin-treated mice after chronic alcohol drinking. Figure 5D showed that insulin treatment greatly increased AKT phosphorylation of Ser473 in the eWAT of Tlr4^LoxTB^ mice. However, eWAT from Tlr4^LoxTB^ × Alb-Cre and Tlr4^LoxTB^ × Zp3-Cre mice showed dramatically reduced p-AKT (Ser473) expression (Figure 5D,E). Regardless of the genotypes, comparably increased p-GSK3β expression was observed in the eWAT following insulin administration (Figure 5D,F).

### 3.6. Hepatocyte TLR4 Regulates Liver Triglyceride Contents in Mice after Excessive Alcohol Intake

**Figure 6:**
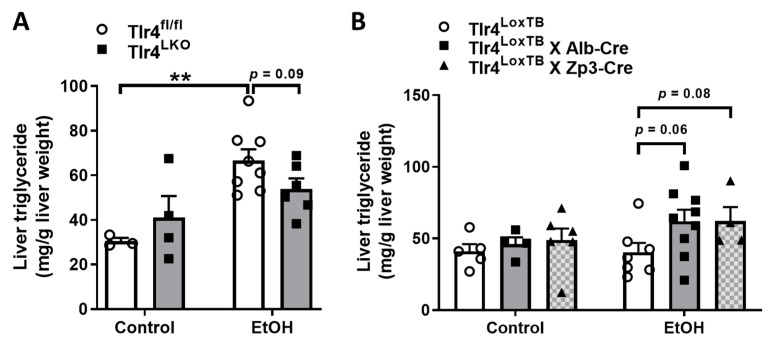
Liver triglyceride contents were examined in mice with TLR4 deleted in hepatocytes (**A**, control treatment (*n* = 3–4) and EtOH treatment (*n* = 6–8)) and mice with TLR4 reactivated in hepatocytes (**B**, control treatment (*n* = 4–6) and EtOH treatment (*n* = 4–9)) after chronic control or alcohol feeding. **, *p* < 0.01, compared between Tlr4^fl/fl^ mice on different diets. All data are presented as mean ± SEM.

It has been widely reported that chronic alcohol administration leads to hepatic steatosis in rodents and human subjects [29]. Consistently, we found that 8 weeks of alcohol intake caused significantly increased liver triglyceride contents in Tlr4^fl/fl^ mice (Figure 6A). However, mice lacking hepatocyte TLR4 tended to accumulate less triglyceride in the liver after chronic alcohol feeding (Figure 6A). Interestingly, global TLR4 knockout mice (Tlr4^LoxTB^) were resistant to alcohol overconsumption-induced hepatic steatosis (Figure 6B). In contrast, reactivation of endogenous TLR4 in hepatocytes and in the whole body promoted more triglyceride accumulation in their livers (Figure 6B). These data suggest that hepatocyte TLR4 plays an important role in regulating chronic alcohol-induced fatty liver development.

## 4. Discussion

The relationship between alcohol consumption and insulin sensitivity is complicated. Accumulating epidemiological studies suggest that light to moderate alcohol intake is associated with enhanced insulin sensitivity [1,30,31,32]; however, heavy drinking promotes the development of insulin resistance [2,30]. Consistent with the impaired insulin action observed in individuals drinking a large amount of alcohol, experimental animals with chronic alcohol intake develop glucose intolerance [4,6,12] and/or insulin resistance [5,6,12]. Carr et al. reported that 4 weeks of chronic alcohol feeding leads to glucose intolerance and insulin resistance in C57BL/6 mice. In addition, significantly increased hepatic glucose production and reduced peripheral glucose disposal were observed in mice fed an alcohol-containing liquid diet for 8 weeks [7]. Collectively, these findings suggest a correlation between excessive alcohol consumption and insulin resistance.

In agreement with previous reports, we also observed that alcohol overconsumption led to reduced glucose tolerance and impaired insulin sensitivity in Tlr4^fl/fl^ mice (Figure 1D,G). Interestingly, alcohol-fed hepatocyte TLR4 deficient mice (Tlr4^LKO^) were insulin sensitive during ITT (Figure 1G) and showed increased insulin-stimulated phosphorylation of GSK3β in the liver (Figure 2A). Taking advantage of the cell-type specific TLR4 reactivatable mouse model (Tlr4^LoxTB^), we generated mice that restore endogenous TLR4 expression only in hepatocytes under TLR4 null background (Tlr4^LoxTB^ × Alb-Cre). This reactivation model could prevent the changes in TLR4 expression in other cell types and limit their potential contributions to disease development. We found that, compared with Tlr4^LoxTB^ mice, Tlr4^LoxTB^ × Alb-Cre mice tended to develop glucose intolerance after chronic alcohol feeding (Figure 4B). In addition, insulin-stimulated AKT phosphorylation was dramatically reduced in the livers of Tlr4^LoxTB^ × Alb-Cre mice following heavy drinking, which was comparable to Tlr4^LoxTB^ × Zp3-Cre mice (Figure 5A). Furthermore, Tlr4^LoxTB^ × Alb-Cre mice exhibited the lowest expression of p-AKT on Ser473 in the eWAT (Figure 5D). These data suggest that hepatocyte TLR4 is sufficient to impair insulin sensitivity in alcohol-fed mice. 

Our IPGTT experiments showed that global TLR4 deficient mice (Tlr4^LoxTB^) following chronic alcohol-containing liquid diet feeding developed systemic glucose intolerance (Figure 4B,C). This is not surprising since it has been reported that mice lacking intestinal epithelial TLR4 showed impaired glucose tolerance after a HFD feeding [33]. Therefore, TLR4 signaling in different cell types may trigger opposite effects on the development of the metabolic syndrome. In contrast to previous findings that adenovirus-mediated overexpression of TLR4 in the liver caused insulin resistance in chow-fed wild-type mice [21], in the current study, similar glucose tolerance and insulin sensitivity were observed in Tlr4^LoxTB^ × Alb-Cre and littermate Tlr4^LoxTB^ mice after chronic control liquid diet feeding (Figure 4A,D). The discrepancy between these two studies could be due to the differences in mouse models and diets. In addition, we cannot rule out the possibility that the combined effect of adenovirus-induced liver injury and TLR4-mediated inflammation could contribute to impaired insulin sensitivity in wild-type mice on a chow diet [21].

Despite the consistent observation that heavy drinking causes systemic insulin resistance in rodents, the discrepancy remains regarding the alterations of proteins involved in insulin signaling pathways. For example, reduced p-AKT expression has been observed in alcohol-exposed mouse livers [4,7,34]. However, He et al. reported increased AKT phosphorylation at Ser473 in the livers of alcohol-fed rats [9]. In eWAT isolated from alcohol-fed mice, both reduced [35] and increased [36] p-AKT protein levels were reported. Moreover, Poirier et al. showed that insulin-stimulated AKT phosphorylation was not affected in alcohol-treated adipocytes [15]. GSK3β is negatively regulated by insulin via AKT phosphorylation. Phosphorylated GSK3β at Ser9 inhibits its kinase activity and enhances insulin-stimulated glycogen synthesis [37,38]. In addition, GSK3β has been shown to directly phosphorylate insulin receptor substrate-1 and impair insulin signaling [37]. Consistent with the concept that alcohol drinking suppresses insulin sensitivity, reduced GSK3β phosphorylation has been reported in the liver and eWAT of alcohol-fed rodents [9,35]. In contrast, increased GSK3β Ser9 phosphorylation was observed in mouse livers after 4 weeks of chronic alcohol feeding [4]. These inconsistent observations could be due to different animal models (rat vs. mouse) and/or experimental conditions (ex vivo vs. in vitro, and acute vs. chronic treatment). In the current study, we examined the protein levels of AKT and GSK3β in alcohol-fed mice after acute insulin stimulation. Interestingly, hepatocyte TLR4 deficient mice showed improved insulin sensitivity and elevated GSK3β Ser9 phosphorylation in the liver after chronic alcohol feeding. While reactivation of TLR4 in hepatocytes decreased AKT Ser473 phosphorylation but did not affect p-GSK3β. It is possible that other TLR4-expressing cells in Tlr4^LKO^ mice could influence insulin signaling molecules and contribute to alcohol-induced insulin resistance.

Global TLR4 knockout mice have been reported to protect against alcohol-induced fatty liver disease [17]. Consistently, we found that Tlr4^LoxTB^ mice, which lack TLR4 in the whole body, are resistant to alcohol-induced hepatic triglyceride accumulation (Figure 6B). Interestingly, reactivation of TLR4 in hepatocytes and in the whole body similarly promoted the development of hepatic steatosis in mice after chronic alcohol feeding (Figure 6B). In contrast, alcohol-fed hepatocyte TLR4 deficient mice tended to accumulate less triglyceride in the liver (Figure 6A). These findings indicate that hepatocyte TLR4 plays a role in mediating alcohol-induced fatty liver disease. In addition, the effect of hepatocyte TLR4 on liver triglyceride contents could partially contribute to its role in altering insulin sensitivity in the context of chronic alcohol drinking.

It has been reported that hepatocyte TLR4 deficient mice have reduced systemic and adipose tissue inflammation after chronic HFD and alcohol-containing liquid diet feeding [19,22]. Considering the relationship between inflammation and insulin resistance [39] and the important role of TLR4 in mediating inflammatory response, we speculate that hepatocyte TLR4 is required in mediating tissue and systemic inflammatory response, and its deficiency is correlated with decreased adipose tissue inflammation and enhanced insulin sensitivity. Furthermore, significant attention has been paid to the critical role of crosstalk between the liver and other tissues in disease development. Several hepatokines have been shown to greatly affect adipose tissue inflammatory response and insulin sensitivity [40]. However, the specific hepatokine(s) that mediate hepatocyte TLR4-induced inflammation and insulin resistance following chronic alcohol feeding are largely unknown and require further investigation.

The limitation of the current study was that a relatively small number of Tlr4^fl/fl^ and Tlr4^LKO^ mice were fed the control liquid diet and used in some experiments. For example, we performed IPGTT and ITT experiments in Tlr4^fl/fl^ and Tlr4^LKO^ mice (*n* = 3–4) after 8 weeks of control liquid diet feeding and found that regardless of the genotypes, mice exhibited similar blood glucose levels. Previously we have reported that Tlr4^fl/fl^ and Tlr4^LKO^ mice following a long-term chow diet feeding responded similarly to intraperitoneal injection of either glucose or insulin [22]. Therefore, under conditions that do not cause diseases, such as control liquid diet or chow diet feeding, TLR4 deletion in hepatocytes does not affect glucose metabolism or insulin sensitivity. In addition, comparable liver triglyceride content was observed in control-fed Tlr4^fl/fl^ and Tlr4^LKO^ mice (*n* = 3–4). Consistently, we have observed that hepatocyte TLR4 deficiency did not influence hepatic triglyceride levels when mice were fed a control liquid diet for 4 weeks or acutely treated with maltose dextrin via oral gavage [19]. Nevertheless, the small sample size of control-fed mice should be considered during data interpretation.

In summary, these findings provide in vivo evidence that hepatocyte TLR4 is a significant contributor to the development of excessive alcohol intake-induced insulin resistance. Targeting hepatocyte TLR4 could have therapeutic potential in the prevention and treatment of metabolic syndrome induced by alcohol overconsumption.

## Data Availability

Not applicable.
